# Structural Prior-Guided Weighted Low-Rank Denoising for Short-Wave Infrared Star Images

**DOI:** 10.3390/s26061980

**Published:** 2026-03-22

**Authors:** Chao Wu, Kefang Wang, Teng Wang, Guanzheng Du, Xiaoyan Li, Fansheng Chen

**Affiliations:** 1 Hangzhou Institute for Advanced Study, University of Chinese Academy of Sciences, Hangzhou 310024, China; 2 University of Chinese Academy of Sciences, Beijing 100049, China; 3 Shanghai Institute of Technical Physics, Chinese Academy of Sciences, Shanghai 200083, China

**Keywords:** infrared image, star image denoising, structural prior weight, weighted low-rank sparse decomposition, residual stripe noise

## Abstract

In ground-based short-wave infrared (SWIR) astronomical observations, temperature drift in the detector readout circuit often introduces nonlinear, spatially non-uniform stripe noise together with Gaussian noise, making weak stellar targets easily submerged and difficult to detect. To address this challenge, we propose a structurally guided weighted low-rank denoising method for infrared star images. Going beyond traditional spatial filtering and standard low-rank decomposition, the proposed method integrates physical priors with mathematical optimization into a unified framework. First, the point spread function (PSF) characteristics of stellar targets are used to construct a hierarchical structural filter, which is further transformed into adaptive prior weights. This design preserves weak-target energy while suppressing noise during iterative optimization. Second, by exploiting the global spatial correlation of the image, residual stripes and the background are jointly modeled as a low-rank component for effective separation. Finally, Bilateral Random Projection (BRP) is introduced to accelerate the weighted soft-thresholding iterations. Experiments on real ground-based observation data, together with ablation studies and sensitivity analyses, demonstrate that the proposed method effectively suppresses structured stripe interference while preserving weak stellar targets under low-SNR conditions. In addition, the acceleration module further improves computational efficiency, making the framework more suitable for practical real-time processing.

## 1. Introduction

Utility of ground-based infrared astronomical detection resides in Space Situational Awareness (SSA), near-Earth asteroid searches, and celestial navigation. Absolute inertial reference bases for optical axis calibration and attitude measurement are provided by stellar targets due to stable photometric properties and precise celestial coordinates [1,2]. Operational constraints in ground-based short-wave infrared (SWIR) tasks stem from detector readout circuit temperature drift. Precise matching of the current response state remains elusive for standard two-point non-uniformity correction strategies. The presence of residual non-linear and non-uniform stripe noise results from this limitation [3,4]. Energy pulses oriented perpendicularly to the stripes characterize the noise in the frequency domain. Spatially, the interference presents as strong directional structural artifacts. Coupling between structural noise and Gaussian noise buries texture-less, distant, and sparse weak star points. This phenomenon represents the primary performance bottleneck for all-weather astronomical detection systems [5].

Instead of strictly separating existing approaches into predefined categories, it is more instructive to examine the practical difficulties encountered in single-frame SWIR star imaging. In many real observations, residual background non-uniformity and stripe artifacts interfere with weak stellar responses. Under such conditions, even computationally simple threshold-based schemes become unstable because selecting a reliable threshold is highly scene-dependent. Weak star signals can be suppressed together with noise, while stripe patterns remain partially visible. Spatial filtering methods such as Top-Hat transforms [6], Local Contrast Measure (LCM) [7], and improved variants including RLCM [8] and MPCM [9] attempt to enhance local contrast for target detection. However, their effectiveness relies on an implicit assumption that the surrounding background varies smoothly. This assumption is often violated in SWIR data, where stripe structures exhibit strong spatial correlation. When low-intensity star responses overlap with clustered noise, the distinction between signal and interference becomes ambiguous, particularly in low-SNR scenes [10]. Beyond spatial filtering, decomposition-based models sometimes mistake vertical structural edges as stripe components, weakening meaningful image details [11]. While guided filters can attenuate high-frequency noise, they inherently risk blurring edges and reduce target energy [12]. Furthermore, morphological operators demand strict geometric priors and therefore lack flexibility in complex backgrounds [13]. Methods relying only on local smoothness constraints often struggle when stripe structures show global correlation in real SWIR star images.

Deep learning has achieved impressive results in astronomical image restoration, particularly in solar reconstruction and deep-space enhancement tasks. Convolutional neural networks (CNNs) and U-Net variants have demonstrated strong representation capability in these settings [14,15,16,17]. Generative Adversarial Networks (GANs) have also been explored to recover fine structural details through adversarial training [18]. Furthermore, recent deep learning architectures have shown potential in complex background modeling and spatial feature extraction. For instance, specific network designs have been proposed to balance spatial distribution and spectral feature extraction [19], while foundation models like HyperSIGMA [20] provide new paradigms for robust representation learning. In the context of infrared small target detection, recent studies incorporate multibranch feature aggregation [21] or unfold traditional optimization models into deep learning frameworks, such as RPCANet [22], to improve interpretability and detection performance. Yet transferring these advances to ground-based SWIR star imaging is not straightforward. Real observational datasets are often limited, and reliable annotations are difficult to obtain. Moreover, many operational systems rely on embedded platforms such as FPGA or DSP hardware, where memory capacity and computational budgets are tightly constrained. Under these practical limitations, deploying large-scale neural networks may not always be feasible, especially for applications requiring stable real-time processing.

Low-rank sparse decomposition (LRSD) provides a structured modeling perspective by representing infrared star images as the superposition of a correlated background and sparse point targets. Methods such as Infrared Patch-Image (IPI) [23] and Robust Principal Component Analysis (RPCA) [24] reformulate the image into globally correlated matrix representations. Tensor-based extensions, including RIPT [25], further exploit non-local self-similarity to enhance structural preservation. Improvements in rank approximation have also been explored through non-convex formulations such as PSTNN [26], while acceleration strategies including WNNM [27] and GoDec [28] reduce computational overhead. Recently, several advanced variations in low-rank and sparse representation have been proposed to further improve background modeling. For instance, novel tensor models guided by adaptive structure tensor indicators [29] and interpretation-weighted sparse methods [30] have demonstrated competitive performance in complex infrared scenarios. Additionally, methods incorporating residual tensor enhancement mechanisms [31] have further improved target extraction capability. Nevertheless, despite these recent advances, most existing formulations are still developed under general mathematical optimization assumptions rather than detector-specific physical characteristics. In SWIR systems, temperature-induced stripe drift and detector non-uniformity introduce structured interference that does not fully conform to ideal low-rank models. When such physical behaviors are not explicitly considered, residual stripe energy may partially migrate into the sparse component, leading to false target responses. Uniform soft-thresholding further limits adaptability to spatial signal variations.

To resolve the conflict between stripe suppression and weak-star preservation—namely, the tendency of aggressive denoising to remove structured noise at the cost of weak stellar signals—we introduce a structurally guided prior. By explicitly constraining the globally correlated residual stripe noise within the low-rank component, the proposed method stabilizes the separation between structured background and sparse targets, even under low-SNR conditions.

At the signal level, we embed the optical point spread function (PSF) into the sparse regularization term via a hierarchical LoG-based operator. This spatial prior adaptively shapes the sparsity penalty during iterations, preserving diffraction-limited star morphology while removing background artifacts. Unlike the hierarchical region sampling strategy used in hyperspectral image classification [32], our hierarchical filter kernel is directly derived from the PSF prior of stellar targets and is designed to construct a physically motivated spatial weighting template for SWIR star image denoising.

Finally, we alleviate the computational bottleneck of conventional low-rank decomposition by incorporating Bilateral Random Projection (BRP) into the update step, substantially reducing processing overhead without sacrificing denoising accuracy.

## 2. Structural Prior-Guided Weighted Low-Rank Denoising Algorithm

In ground-based short-wave infrared (SWIR) observations, stellar targets are jointly affected by atmospheric turbulence, optical diffraction, and detector imaging characteristics. As a result, they usually appear as dim, small, and sparsely distributed local spots in the image. Their energy distribution generally follows the modulation pattern of the point spread function (PSF) and occupies only a few pixels, which makes them highly susceptible to background interference. At the same time, due to temperature drift in the detector readout circuit and response inconsistency among pixels, raw SWIR images are often contaminated by pronounced non-uniform stripe noise. Such stripes exhibit strong row- or column-wise correlations in the spatial domain and possess obvious structural characteristics. In addition, the image background is further corrupted by random Gaussian noise and thermal noise, which further increases the difficulty of weak target extraction.

These target and background characteristics together define the main challenge of the task. On the one hand, structured stripe noise can easily obscure weak stellar responses and reduce their separability from the background. On the other hand, conventional denoising methods often suppress target energy while removing stripes, causing weak targets to fade or even disappear. Therefore, the key issue is how to suppress structured background interference while preserving weak stellar targets as much as possible.

**Figure 1:**
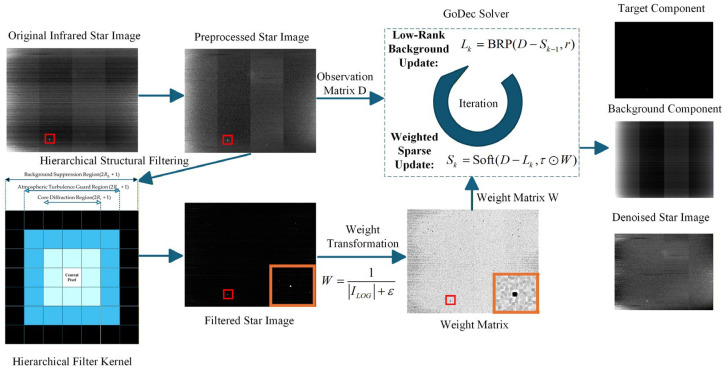
The overall workflow of the proposed algorithm. The red boxes indicate the spatial location of the selected weak stellar target, while the orange boxes provide a magnified view of the corresponding target region to illustrate the effect of structural weight transformation. Within the GoDec solver, the mathematical symbols $L_{k}$ and $S_{k}$ denote the low-rank background update via Bilateral Random Projection and the sparse target update via weighted soft-thresholding, respectively.

To address this problem, we develop a structural-prior-guided weighted low-rank denoising framework. The basic idea is to first reduce non-uniform detector response through radiometric calibration, and then construct prior weights from the PSF-related characteristics of stellar targets to guide the subsequent low-rank and sparse decomposition. The overall processing pipeline is illustrated in Figure 1.

### 2.1. Star Image Preprocessing

Raw SWIR images typically suffer from significant non-uniform stripes and blind pixels due to manufacturing limitations and response discrepancies in the readout circuit column amplifiers. These artifacts severely degrade the signal-to-noise ratio (SNR) of the images and hinder the subsequent extraction of weak stellar signals. Consequently, a standard radiometric calibration procedure is applied prior to the denoising process.

To mitigate stripe noise, a linear model is employed to calibrate the detector response. Within a specific time interval, the relationship between the calibrated output image Y(i,j) and the raw input image X(i,j) for the (i,j)-th pixel is modeled as

(1) Y(i,j)=K(i,j)×X(i,j)+B(i,j).

To determine these model parameters, assuming the pixel response follows a linear law, uniform radiation data from a blackbody are collected at both high and low temperature points to obtain the average focal plane responses, $Y_{L}$ and $Y_{H}$:

(2) $$\left\{\begin{array}{l} Y_{L}\;=\;K(i,j)\times X_{L}(i,j)\;+\;B(i,j)\\ Y_{H}\;=\;K(i,j)\times X_{H}(i,j)\;+\;B(i,j) \end{array}\right.$$

Based on these two sets of calibration data, the gain coefficient K(i,j) and bias coefficient B(i,j) can be calculated as

(3) $$K(i,j)=\frac{Y_{H}-Y_{L}}{X_{H}(i,j)-X_{L}(i,j)}$$

(4) $$B(i,j)=Y_{H}(i,j)-K(i,j)\times X_{H}(i,j)$$

For real-time captured observation images X, the calibrated output is obtained by substituting the pre-calculated coefficients from Equations (3) and (4) into Equation (1).

**Figure 2:**
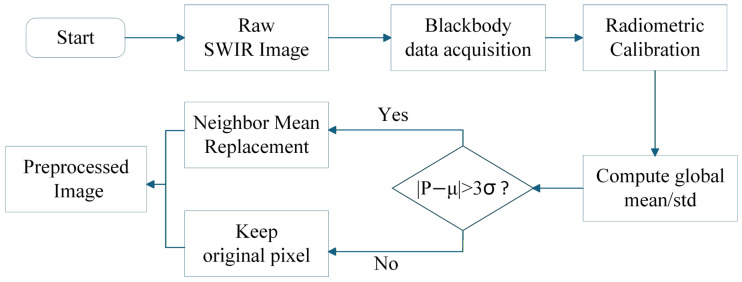
Flowchart of the preprocessing procedure, including radiometric calibration and blind-pixel correction.

Regarding blind pixels, their response characteristics typically exhibit abnormal dynamic ranges and severe non-linearity, fundamentally differing from the linear response of normal pixels. Because the background of the non-uniformity-corrected infrared star images generally follows a normal distribution, the three-sigma (3σ) rule is adopted for outlier removal. Specifically, a pixel is identified as a blind pixel if its grayscale value deviates from the global mean by more than three standard deviations. To ensure local image continuity, the detected blind pixels are compensated using a neighborhood substitution method, where the outlier is replaced by the mean grayscale value of the valid pixels within its four-pixel neighborhood. The complete preprocessing workflow is illustrated in Figure 2.

### 2.2. Global Low-Rank Sparse Decomposition Model

Conventional infrared small target detection and denoising methods often employ tensor or matrix models based on image patches. While these methods exploit local correlation to some extent, they frequently destroy the global structural information of the image and involve high computational redundancy, making it difficult to meet real-time requirements. To address these issues, we propose a full-image low-rank sparse decomposition model that directly mines the global spatial correlation of the background at the original image scale.

**Figure 3:**
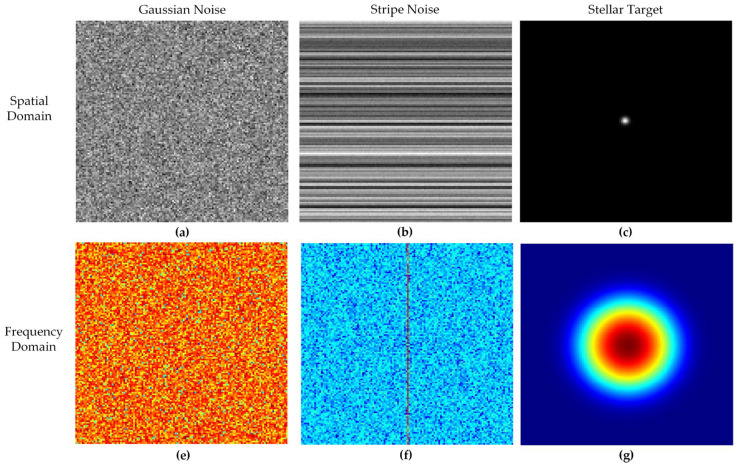
Comparison of spatial and frequency domain characteristics for typical infrared star image components: (**a**) spatial domain of Gaussian noise; (**b**) spatial domain of stripe noise; (**c**) spatial domain of the stellar target; (**e**) frequency domain of Gaussian noise; (**f**) frequency domain of stripe noise; (**g**) frequency domain of the stellar target. For (**e**–**g**), colors indicate normalized spectral energy (red for high, blue for low). The red vertical line in (**f**) represents the concentrated energy of the horizontal stripes.

A single-frame infrared star image can be mathematically represented as a matrix $D\in {\mathbb{R}}^{m\times n}$. Specifically, D is directly formed by assembling all the preprocessed pixels $Y_{i,j}$ generated from Equation (1). By feeding the radiometrically calibrated image into the decomposition model, the image is modeled as a linear superposition of a low-rank background, sparse targets, and Gaussian noise:

(5) D = L + S + G

where L, S and G represent the low-rank background, sparse targets, and Gaussian noise, respectively. To reveal the mechanism of this physical model in handling residual stripes and weak signals, we perform a comparative analysis of each component from two dimensions: spatial features and frequency response, as illustrated in Figure 3. To intuitively demonstrate the structural differences among the image components, the visualizations in Figure 3 are computational simulations generated based on the mathematical models of infrared sensors. Specifically, the visualizations are computed within a 30×30 spatial grid. The stellar target is simulated using a 2D Gaussian function with a standard deviation of σ=2 pixels. The non-uniform stripe noise is modeled by generating highly correlated random row vectors to mimic readout circuit drift. The background noise is drawn from a zero-mean normal distribution. The corresponding frequency domain representations are computed via the 2D Fast Fourier Transform (FFT).

- Low-Rank Component (L): This component corresponds to the non-uniform residual stripes caused by temperature drift in the SWIR detector’s readout circuit. As shown in Figure 3b, these stripes exhibit significant spatial row (or column) correlations. In the frequency domain (Figure 3f), their energy is intensely compressed into axial pulses passing through the spectrum center. This low spatial degree of freedom strictly satisfies the low-rank assumption (rank(L)≤r), providing the theoretical premise for utilizing low-rank matrix approximation to strip away hardware-level physical defects.
- Sparse Component (S): This corresponds to the weak stellar targets modulated by the optical system’s Point Spread Function (PSF). In the spatial domain (Figure 3c), these targets show up as isolated bright spots that take up barely any pixels, clearly proving their high spatial sparsity. Meanwhile, in the frequency domain (Figure 3g), they look like isotropic circular spots that smoothly fade outward. These specific morphological traits are utilized in the algorithm design. The preservation of the original target energy is strictly maintained during the iteration process.
- Noise Component (G): Detector thermal noise and shot noise constitute the primary sources of this component. Random grayscale fluctuations characterize the Gaussian noise in the spatial domain (Figure 3a). A uniform energy distribution across all frequency bands is exhibited in the frequency domain (Figure 3e).

These component characteristics are directly reflected in the subsequent processing. Specifically, the stripe-dominated background is modeled as the low-rank component L and suppressed through low-rank regularization, the weak stellar targets are modeled as the sparse component S and preserved by the PSF-guided weighting strategy, and the random background fluctuations are modeled as the Gaussian noise component G and treated as residual disturbance during optimization.

Denoising in ground-based infrared star images is fundamentally modeled as a dual-constraint optimization task derived from the identified physical attributes. Structural stripe removal is achieved via low-rank constraints targeting high-energy concentrations. Precision capture of isotropic weak targets within wideband Gaussian backgrounds is simultaneously facilitated through the integration of PSF-based prior weights.

### 2.3. Construction of Structural Prior Weights

Traditional GoDec algorithms usually start with the assumption that sparse elements are distributed uniformly across the entire image. However, in ground-based infrared star images with low signal-to-noise ratios (SNR), weak stellar targets are often submerged in intense noise and residual stripes. Under globally uniform sparse regularization, this leads to excessive shrinkage of target energy or the misidentification of weak targets as noise. To solve this problem, we decided to move past the limitations of standard morphological filters. Instead, we developed a structural weight matrix centered on the Point Spread Function (PSF) prior. This allows our model to protect targets precisely while suppressing the background by assigning physical energy weights based on spatial characteristics.

**Figure 4:**
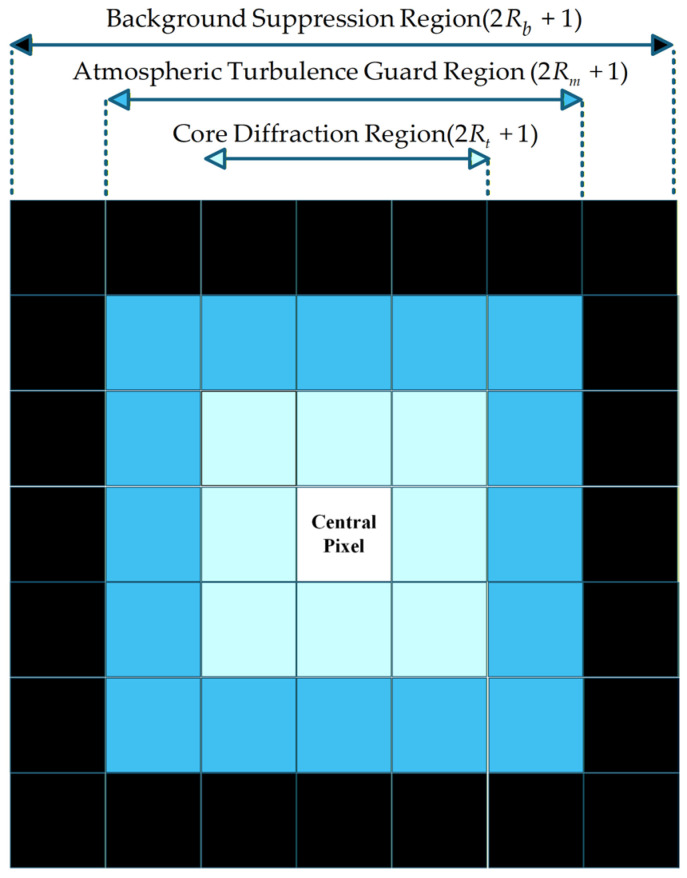
Schematic diagram of hierarchical rectangular regions. The white central region denotes the Core Diffraction Region, the light blue region denotes the Atmospheric Turbulence Guard Region, and the dark blue/black peripheral region denotes the Background Suppression Region.

In ground-based astronomical observation, stellar targets are modulated by both the optical system diffraction limit and atmospheric seeing. Consequently, their energy distribution on the focal plane approximates a 2D Gaussian diffusion model. Based on this prior, a hierarchical structure-guided Laplacian of Gaussian (LoG) spatial response model is constructed, as illustrated in Figure 4. In this way, specific spatial dimensions and weight responses are defined to align with the grayscale characteristics of infrared stars. We define the hierarchical convolution kernel K(x,y) for a pixel located at a Euclidean distance $d=\sqrt{x^{2}+y^{2}}$ from the center in the following way:

(6) $$K(x,y)=\left\{\begin{array}{ll} LoG(x,y|\sigma ), & if\;d\leq R_{t}\\ 0, & if\;R_{t}<d\leq R_{m}\\ -1, & if\;R_{m}<d\leq R_{b} \end{array}\right.$$

Instead of relying on empirical guesses, we directly link the scale parameter σ to the actual Gaussian blur radius $\sigma_{PSF}$ of our SWIR detection system. We define each region based on specific design criteria and physical meanings:

- Core Diffraction Region ($d\leq R_{t}$): This region focuses on capturing the vast majority of stellar energy and is assigned a standard LoG positive response. Based on the Airy disk radius in the observed waveband and the detector pixel size, $R_{t}$ is dynamically set to enclose >95% of the diffused energy. This physical constraint ensures that the operator maximizes its response to real star points conforming to the diffraction-limited morphology. The physical mapping relationship is given by:
  (7) $$R_{t}=\left\lceil \frac{1.22\times \lambda \times F}{p}\right\rceil$$
  where λ represents the central observation wavelength, F is the focal ratio of the optical system, p denotes the pixel size of the SWIR detector, and $\left\lceil ∙\right\rceil$ is the ceiling operation.
- Atmospheric Turbulence Buffer Region ($R_{t}<d\leq R_{m}$): Located between the star and the background, this region is assigned a zero weight response. Physically, it establishes an energy isolation band to accommodate edge energy jitter and gradient diffusion caused by time-varying atmospheric turbulence during ground-based observations. This guard region effectively prevents the high-frequency dispersed energy of the star from being suppressed by peripheral mechanisms, guaranteeing grayscale integrity.
- Background Suppression Region ($R_{m}<d\leq R_{b}$): This region estimates local spatial baseline noise and is assigned a negative constant weight (e.g., fundamentally−1). By utilizing the negative response of the outer region to actively cancel the DC baseline component of the inner region, this “positive-center, negative-periphery” differential mechanism effectively strips away low-frequency interference, such as large-scale cloud backgrounds and non-uniform residual stripes.

First, the hierarchical operator is convolved with the preprocessed image matrix $X_{in}$ to obtain the saliency map $I_{LoG}$. In this map, real weak stars exhibit exceptionally high positive energy responses due to their spatial compliance with the PSF, while residual stripes and shot noise are significantly suppressed as they do not conform to Gaussian morphological features. It should be emphasized that the larger kernel regions are not introduced for simple wide-area smoothing. Instead, the hierarchical kernel assigns different responses to different spatial zones. The central positive region preserves PSF-consistent target energy, the intermediate zero-response band avoids suppressing turbulence-induced edge diffusion, and the outer negative region suppresses large-scale background fluctuations and residual stripe components. Therefore, increasing the kernel size does not simply accumulate more noise; rather, it improves the separability between weak stellar targets and structured background through a spatially differential design. To transform this saliency map into the penalty weight matrix W required for optimization, an inverse mapping strategy is employed:

(8) $$W_{ij}=\frac{1}{1+k\times N(I_{pos}(i,j))+\varepsilon }$$

where N(∙) denotes the linear normalization operation, $I_{pos}=max(I_{LoG},0)$ retains only the physical positive responses, k is the contrast enhancement factor, and ε is a small constant to prevent division by zero. As indicated, the weight $W_{i,j}$ is inversely related to the PSF saliency response. In regions containing stars, the strong LoG response results in a minimal weight $W_{ij}$. This minimizes the sparse penalty for these regions during the subsequent GoDec iterations, thereby effectively preserving the target energy. By accurately indicating target locations that match optical priors, this weight matrix alleviates the limitations of traditional blind mathematical optimization by introducing physically informed constraints.

Different from conventional weighted sparse decomposition strategies, where weighting factors are typically constructed based on empirical rules or statistical intensity distributions, the proposed weight matrix is derived from the physical PSF characteristics and optical diffraction constraints of the SWIR imaging system. This establishes a direct linkage between imaging formation physics and optimization regularization, ensuring structural consistency during sparse recovery. Therefore, the proposed weighting mechanism is not merely a mathematical adjustment, but a physically grounded regularization design tailored to SWIR imaging characteristics.

The PSF-related parameters are not heuristically tuned but analytically computed from measurable optical specifications, such as the central wavelength, focal ratio, and detector pixel size. When the optical configuration changes, the corresponding parameters can be recalculated directly according to these physical quantities, rather than manually adjusted. This ensures that the proposed weighting mechanism maintains physical consistency and practical transferability across different SWIR imaging systems.

### 2.4. Accelerated Weighted GoDec Optimization Solver

To integrate the structural prior weights constructed in Section 2.3 into the decomposition process and overcome the limitations of the traditional GoDec algorithm—which tends to lose weak targets due to hard-thresholding truncation under low signal-to-noise ratio (SNR) conditions—we propose an improved weighted GoDec model. Its optimization objective function is defined as

(9) $$\underset{L,S}{min}\frac{1}{2}\|D-L-S{\|}_{F}^{2}+\tau \|W⊙S{\|}_{1}\;s.t.\;rank(L)\leq r$$

where τ is the sparse regularization parameter, and $\|W⊙S{\|}_{1}$ denotes the weighted $L_{1}$ norm. This non-convex optimization problem is solved by updating the low-rank component L and the sparse component S through alternating iterations, primarily comprising the following two core steps, as summarized in Algorithm 1:

- Low-Rank Background Update: To avoid direct Singular Value Decomposition (SVD) on large-scale matrices, we introduce the Bilateral Random Projection (BRP) technique to approximate the low-rank matrix. In the k-th iteration, the background $L_{k}$ is updated using the residual $D-S_{k-1}$ from the previous round:
  (10) $$L_{k}=BRP(D-S_{k-1},r)$$
  where r is the predefined target rank of the background matrix, which controls the spatial degree of freedom for the low-rank approximation. By employing the BRP algorithm, the computational complexity of the low-rank update is significantly reduced from $O(mn^{2})$ of the standard Singular Value Decomposition (SVD) used in the baseline Robust Principal Component Analysis (RPCA) method to O(mnr) (where r≪min(m,n)). This fundamental algorithmic acceleration enables millisecond-level background fitting.
- Weighted Sparse Update: When extracting sparse targets, the structural weight matrix W is utilized to adaptively adjust the penalty threshold. The sparse component $S_{k}$ in the k-th iteration is solved via the weighted soft-thresholding operator:
  (11) $$S_{k}=\Gamma_{\tau ⊙W}(D-L_{k})$$
  where ⊙ represents the Hadamard (element-wise) product, Γ(∙) is the soft-thresholding operator, and τ serves as the base threshold coefficient. The core mechanism of this step is that in stellar regions, the exceptionally small value of W significantly lowers the actual threshold, thereby prioritizing the preservation of weak target energy. Conversely, in background noise regions, the large value of W raises the threshold, ensuring thorough noise filtration.

Upon reaching convergence through these alternating iterations, the observation matrix D is decomposed into a low-rank background L, sparse targets S, and random Gaussian noise G. To achieve the ultimate goal of target enhancement and noise suppression, we discard the structured background component containing residual stripes and directly output the sparse component as the final denoised star image (i.e., $I_{clean}=S$).
**Algorithm 1:** Accelerated Weighted GoDec Solver**Input:** Infrared star image observation matrix D, background rank r, base threshold coefficient τ, prior weight matrix W, maximum iterations K, convergence tolerance ε.**Initialization:** $L_{0}=D$, $S_{0}=0$, k=0.**While** $\|D-L_{k}-S_{k}{\|}_{F}^{2}/\|D{\|}_{F}^{2}>\varepsilon$ **do**  Update iteration counter: k←k+1;  //Step 1: Low-rank background update (BRP acceleration)  Calculate background residual: $E_{L}=D-S_{k-1}$;  Update background matrix: $L_{k}=BRP(E_{L},r)$;  //Step 2: Sparse target update (Weighted soft-thresholding)  Calculate target residual: $E_{S}=D-L_{k}$;  Update target matrix: $S_{k}=\Gamma_{\tau ⊙W}(E_{S})=sign(E_{S})∙max(|E_{S}|-\tau ⊙W,0)$;End While**Output:** Low-rank background L, sparse targets S.

## 3. Experimental Results and Analysis

### 3.1. Experimental Platform and Data Acquisition

**Figure 5:**
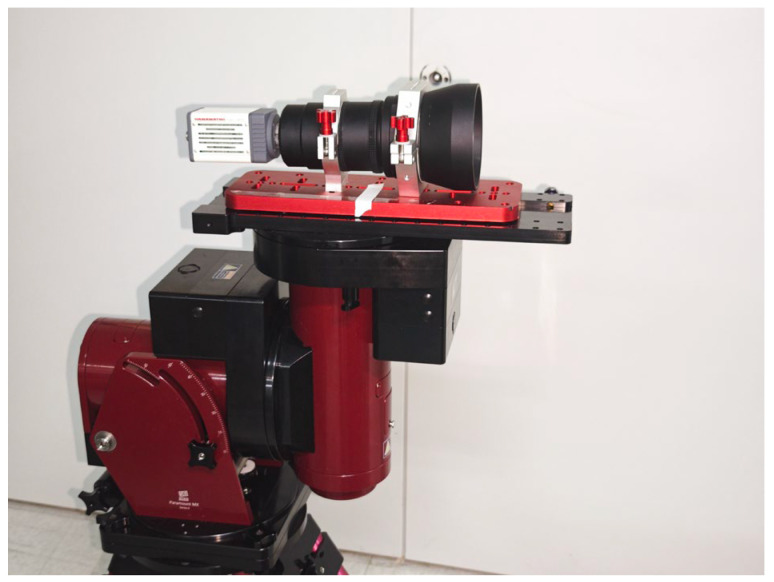
Experimental platform for SWIR star image acquisition.

To evaluate the proposed algorithm under real observation conditions, we built a ground-based SWIR imaging platform, as shown in Figure 5. The system consists of a SWIR camera with an InGaAs sensor and an infrared optical lens, both mounted on a high-precision equatorial platform to ensure stable star tracking. The main hardware specifications are summarized in Table 1. Data collection was performed at 20:00 on 11 December 2024. The exposure time was set to 16.7 ms, which corresponds to the hardware limit of the camera and minimizes dark current accumulation in the detector. Five real SWIR star-image sequences were used in the experiments, and each sequence contains 100 consecutive frames acquired under continuous observation. The selected sequences span different SNR levels, allowing performance comparison under varying observation conditions.

Based on the core optical parameters listed in Table 1, the focal ratio F of the system is approximately 2.41. Assuming a typical central wavelength of λ=1.5 μm for the SWIR band, the theoretical Airy disk diffraction radius of a stellar target on the focal plane is calculated to be 4.41 μm according to Equation (7). Since this physical diffraction scale is significantly smaller than the detector’s single pixel size (20 μm), its corresponding theoretical radius on the image is merely 0.22 pixels. Accounting for the slight energy broadening caused by atmospheric seeing during actual ground-based observations, a ceiling operation is applied to this pixel radius. Consequently, the radius of the core diffraction region can be theoretically derived as $R_{t}=1$, which precisely corresponds to a 3 × 3 pixel neighborhood in the spatial domain.

### 3.2. Results and Analysis

To evaluate the performance of the proposed algorithm, we conducted comparative experiments against four representative baseline methods: the Top-Hat transform, Laplacian of Gaussian (LoG) filter, classical Robust Principal Component Analysis (RPCA), and Joint Local Contrast Measure (JLCM) [33]. All algorithms were implemented in MATLAB R2022a on a workstation equipped with an Intel Core i9-13980HX CPU and 16 GB RAM. To ensure a fair comparison, all methods were applied to the same set of raw observations after the standardized radiometric calibration and blind-pixel correction described in Section 2.1.

**Figure 6:**
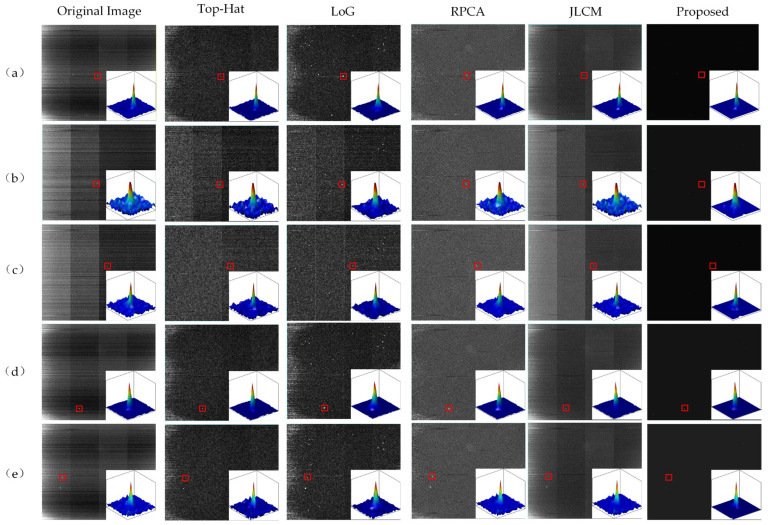
Comparison of results before and after denoising on real infrared star images. Rows (**a**–**e**) correspond to the target regions of HIP 28380, HIP 50335, HIP 50583, HIP 11767, and HIP 24608 sequences, respectively. The columns represent different denoising algorithms as labeled at the top. The red boxes indicate the spatial locations of the star targets. The magnified 3D insets illustrate the grayscale distribution of the targets, where different colors represent the normalized intensity levels (with red indicating peak intensity and blue indicating background).

**Figure 7:**
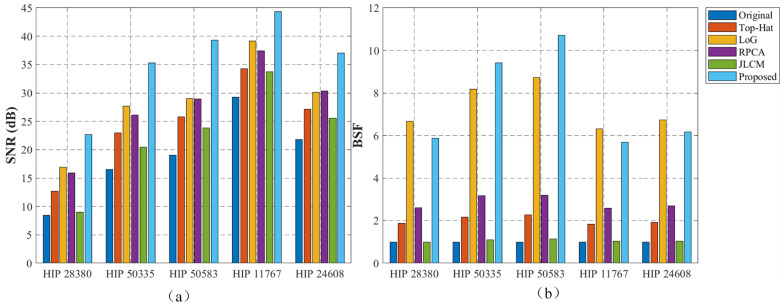
Distribution of quantitative metrics across different SWIR star image sequences: (**a**) Comparison of SNR performance; (**b**) Comparison of BSF performance.

The test dataset consists of five real SWIR video sequences (HIP 28380, HIP 50335, HIP 50583, HIP 11767, and HIP 24608). For each sequence, 100 consecutive frames were available for observation, and the quantitative comparison in Table 2 is reported on representative single-frame results. The quantitative evaluation relies on three standard metrics: Signal-to-Clutter Ratio (SCR) measures the local contrast of the target against its nearby background; Signal-to-Noise Ratio (SNR) reflects the target’s signal strength relative to the global image noise; and Background Suppression Factor (BSF) is calculated as the ratio of the background standard deviation before and after processing, directly indicating how well the stripes and clutter are removed. Representative visual results for single frames are shown in Figure 6. The comprehensive quantitative comparisons are listed in Table 2 and intuitively visualized in Figure 7.

Detection capability under low SNR conditions is a crucial metric for evaluating algorithmic performance. Taking the extreme detection scenario in the HIP 28380 sequence as an example—where the target signal is extremely weak (SNR of only 8.48 dB) and completely submerged in intense Gaussian noise—the data in Table 2 reveal several limitations of traditional methods. Limited by the fixed scale of its structuring elements, the traditional Top-Hat transform struggles to identify the morphological features of the target, often misidentifying high-intensity single-pixel noise as stars. Although the LoG filter possesses edge enhancement capabilities, its isotropic operator is highly sensitive to high-frequency random noise; it amplifies background Gaussian noise while enhancing the target, which severely restricts the improvement of the output SNR. Consequently, the SCR metrics of these two traditional algorithms (9.87 dB and 8.27 dB, respectively) are even lower than that of the original image (10.35 dB). This indicates that traditional filtering methods severely deplete the energy of weak targets while removing noise, making them highly prone to missed detections. In contrast, the structural prior weight matrix introduced in the proposed method utilizes the unique Point Spread Function (PSF) characteristics of stars to accurately distinguish sparse targets from randomly distributed Gaussian noise, applying heavy penalties to random noise components lacking structural features. Experimental results demonstrate that while effectively filtering out Gaussian noise, the proposed algorithm successfully reconstructs the grayscale of weak stars, elevating the SNR from 8.48 dB to 22.69 dB, and the SCR to 11.62 dB. The 3D energy maps in Figure 6 present a clearer separation between background responses and star-related impulses. Even under this extreme low-SNR condition, the weak stellar structure is still observable rather than being overwhelmed by noise.

Persistent time-varying and spatially non-uniform stripe noise remains a major difficulty in SWIR star imaging. In the Top-Hat method, once the stripe scale exceeds the predefined structuring element, background estimation becomes unstable. For RPCA-IALM, stripe intensity fluctuations increase the effective rank of the background matrix, which weakens the low-rank assumption and causes part of the structured energy to enter the sparse component. The JLCM method mainly relies on local contrast and does not impose a global structural constraint, so its BSF stays close to 1.0. After incorporating structural prior weighting into the decomposition process, the separation becomes more stable across stripe variations. In the HIP 50583 sequence, the BSF reaches 10.70, compared with 3.20 for RPCA and 1.13 for JLCM. The stripe pattern in the recovered image is visibly weakened, and background fluctuation is reduced.

As shown in Table 3, classical spatial filters (Top-Hat, LoG) and local contrast methods (JLCM) possess an inherent computational speed advantage, typically completing execution within a few milliseconds. However, this high computational speed comes at the severe cost of detection performance; as previously demonstrated in Table 2, these methods suffer from severe background residue and fail to effectively preserve the energy of weak stellar targets. In contrast, the baseline RPCA method achieves effective background suppression, but its repeated Singular Value Decomposition (SVD) operations create a computational bottleneck, taking 1.37 s per frame in our experimental environment. By introducing the Bilateral Random Projection (BRP) technique, the proposed method bypasses the full SVD calculation and directly resolves this bottleneck, drastically reducing the processing time to 0.16 s—achieving an 8.56 speedup compared to traditional RPCA. Overall, although our method requires more computational time than simple spatial filters, it provides excellent target enhancement within a fully acceptable time cost, yielding high engineering practicality for real-world ground-based observation systems.

### 3.3. Ablation Study on the Core Algorithm

To better understand the contribution of the structural prior weight within the low-rank sparse framework, we conducted a comparative ablation experiment. The baseline decomposition model without prior weighting (Standard Low-Rank) was tested against two weighted versions: a standard Gaussian weighted model and the proposed PSF-weighted model. Prior to any decomposition, all sequences were strictly preprocessed (as detailed in Section 2.1) under identical conditions. This setup allows us to focus specifically on how different weighting strategies influence weak target retention. Unlike conventional weighted sparse models where weights are heuristically designed or statistically estimated, the proposed weight matrix is derived from the physical PSF characteristics and optical diffraction constraints of the SWIR system. This establishes a direct physical consistency between imaging formation and optimization regularization.

**Figure 8:**
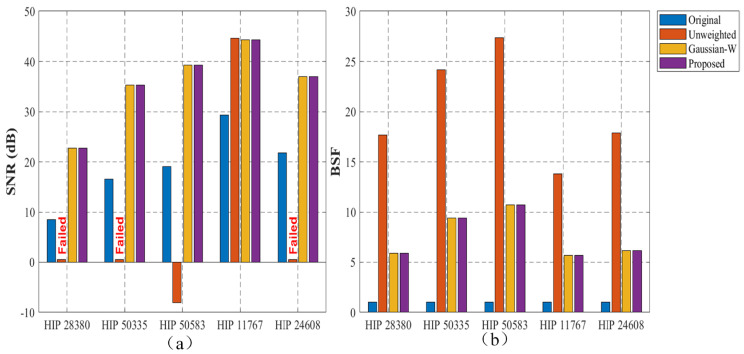
Distribution of quantitative metrics in the ablation study: (**a**) SNR performance highlighting the failure cases of the unweighted model; (**b**) BSF performance.

Under extremely low-SNR conditions (e.g., HIP 28380, HIP 50335, and HIP 24608), the standard low-rank baseline fails to retain weak star signals. As visually demonstrated in Figure 8 and quantitatively listed in Table 4, without structural constraints, weak targets are frequently misclassified as sparse noise and eliminated during globally uniform soft-thresholding, resulting in failed SNR and SCR measurements. In more complex background scenarios such as HIP 50583, the baseline model produces a negative SNR (−8.06 dB), indicating severe background leakage. This behavior demonstrates that uniform thresholding cannot adapt to spatial signal variations.

To verify whether a general mathematical weight is sufficient to solve this issue, we evaluated the Gaussian weighted model. While the Gaussian weighting strategy provides basic spatial constraints and successfully recovers the submerged targets—achieving an SNR of 16.37 dB for HIP 28380 and 32.62 dB for HIP 50583—its enhancement capability is limited. Because it relies merely on a standard statistical distribution without explicit physical boundaries, such as the atmospheric turbulence buffer region and the negative background suppression region defined in our method, it cannot optimally separate the target from residual stripes.

In contrast, after introducing the proposed structural prior weight, signal recovery becomes significantly more stable and accurate. The proposed weighting strategy effectively suppresses structured background interference and drastically improves the SNR to 22.69 dB for HIP 28380 and 39.31 dB for HIP 50583, outperforming the Gaussian weight by over 6 dB. Across different sequences, the proposed model shows the most stable behavior, particularly in low-SNR conditions. Overall, incorporating structural prior information leads to more consistent preservation of sparse star responses than both unweighted and conventionally weighted models across different observation scenarios.

We also evaluated the role of the proposed Bilateral Random Projection (BRP) module from an ablation perspective. Since the overall runtime comparison with other representative methods has already been presented in Table 3, the purpose here is not to repeat a cross-method efficiency analysis, but to verify whether BRP affects the reconstruction quality of the proposed framework itself. For this reason, we compared the complete method with an unaccelerated version that uses standard Singular Value Decomposition (SVD) for the low-rank update. The results show that introducing BRP does not change the recovered detection metrics, while reducing the average per-frame processing time from 3.90 s to 0.22 s. This indicates that BRP functions as an effective acceleration module that improves solver efficiency without altering the detection performance.

### 3.4. Parameter Sensitivity and Failure Mode Analysis

To evaluate the robustness of the proposed method, we analyzed the sensitivity of the key hyperparameters in the weight transformation and identified potential failure modes based on our modeling assumptions.

**Figure 9:**
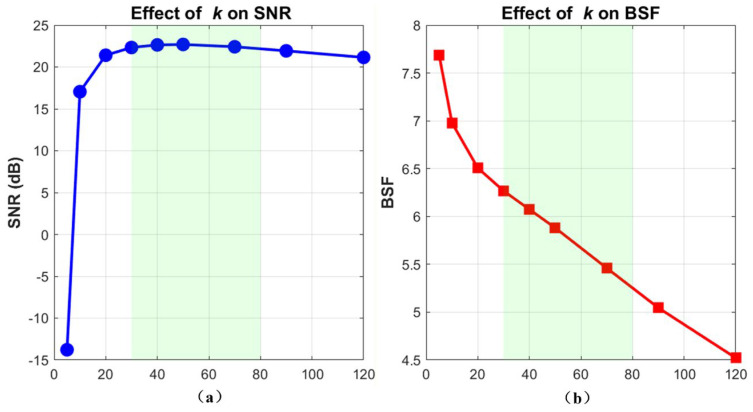
Sensitivity of the proposed method to the contrast enhancement factor k: (**a**) effect of k on SNR; (**b**) effect of k on BSF.

The contrast enhancement factor k determines the separation margin between targets and the background. As shown in Figure 9a, a small k fails to provide sufficient weight disparity, causing weak stellar signals to be erroneously suppressed alongside the noise. For instance, on the extremely low-SNR HIP 28380 sequence, SNR drops sharply to −13.77 dB at k=5. However, once k enters a moderate range (k≥30), SNR stabilizes around 22.69 dB, proving that the algorithm is robust to parameter variations within this plateau. Meanwhile, Figure 9b illustrates that BSF gradually decreases as k increases. This is a reasonable physical trade-off: a larger k provides stronger protection for diffraction-limited structures, which inherently retains some local high-frequency residual noise around the target. To balance target fidelity and background suppression, we adopted k=50 as the default setting for all experiments.

**Figure 10:**
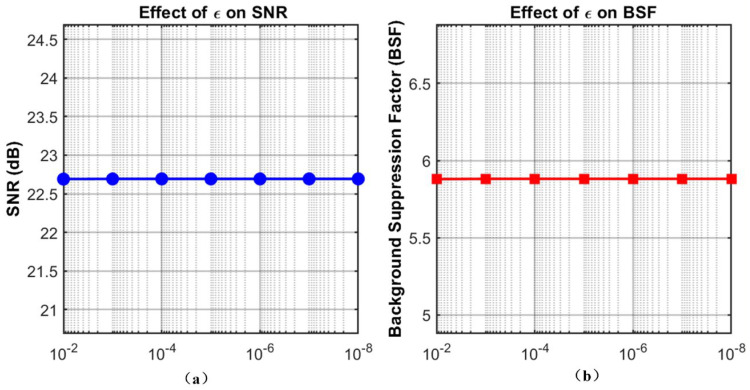
Sensitivity of the proposed method to the small constant ϵ: (**a**) effect of ϵ on SNR; (**b**) effect of ϵ on BSF.

The small constant ϵ, conversely, serves purely to prevent division-by-zero errors. As shown in Figure 10, varying ϵ from $10^{-2}$ to $10^{-8}$ yields absolutely no fluctuation in either SNR or BSF. This confirms that the final reconstruction is completely insensitive to ϵ, eliminating the need for manual tuning across different datasets.

Our method is built on the assumptions underlying the structural prior and low-rank model. When these assumptions are not satisfied in real observations, the denoising performance may degrade. One representative failure case is severe optical defocus. In this situation, the stellar footprint may deviate significantly from the assumed diffraction-limited Gaussian profile, which reduces the accuracy of the structural prior. As a result, the weighting strategy may fail to properly emphasize the target region, and part of the valid signal may be absorbed during the decomposition process. Another challenging case arises in extremely dense star clusters, where strong target overlap violates the sparsity assumption. When multiple stars are closely packed, they may form locally correlated structures that are difficult to distinguish from the background, causing incomplete separation between target and background components. Therefore, the proposed method is more suitable for scenes in which the stellar shape remains relatively stable and the targets are spatially separable. These extreme cases were not encountered in the current dataset and will be investigated further in future work.

Overall, the proposed method is effective in suppressing structured stripe background while preserving weak stellar targets under low-SNR conditions, and the BRP module further improves computational efficiency. However, the method still relies on the assumed target morphology and sparsity prior. Its performance may degrade when severe optical defocus causes the stellar footprint to deviate from the assumed PSF, or when dense target overlap weakens the sparsity assumption.

## 4. Conclusions

Ground-based SWIR imagery poses a major challenge for weak-star extraction because persistent stripe interference is often coupled with severe low-SNR conditions. To address this problem, we proposed a structurally guided weighted low-rank framework. By embedding PSF-related physical priors into sparse regularization, the method prevents dim stellar responses from being erroneously absorbed into the background. In addition, replacing standard SVD with Bilateral Random Projection in the low-rank update substantially improves computational efficiency and makes real-time deployment more practical. Experimental, ablation, and sensitivity results collectively demonstrate that the proposed framework achieves stable single-frame weak-star recovery without relying on complex multi-frame registration, making it promising for deployment on resource-limited ground-based systems. Nevertheless, the method still relies on assumptions about target morphology and sparsity, and its performance may degrade under severe optical defocus or extremely dense star clusters. These limitations will be addressed in future work through more adaptive prior modeling.

## Figures and Tables

**Table 1:** Key performance parameters of the experimental hardware system.

System Device	Parameter	Value
Infrared lens	Focal length	200 mm
	Aperture	83 mm
SWIR camera	Pixel size	20 × 20 μm
	Resolution	640 × 512
	Waveband	0.9–1.7 μm
	Exposure time	16.7 ms
Equatorial mount	Load capacity	113 kg
	Tracking accuracy	$30^{\prime \prime }$

**Table 2:** Quantitative comparison of different algorithms in terms of SCR, SNR, and BSF.

Image	Metric	Original	Top-Hat	LoG	RPCA	JLCM	Proposed
HIP 28380	SCR (dB)	10.35	9.87	8.27	10.91	8.12	**11.62**
	SNR (dB)	8.48	12.69	16.94	15.91	8.98	**22.69**
	BSF	1.00	1.99	6.66	2.61	1.01	**5.88**
HIP 50335	SCR (dB)	21.18	22.06	25.89	24.64	22.39	**34.00**
	SNR (dB)	15.97	22.99	27.67	26.15	20.47	**35.34**
	BSF	1.00	2.17	8.18	3.17	1.10	**9.41**
HIP 50583	SCR (dB)	25.20	26.32	29.14	29.24	27.33	**39.47**
	SNR (dB)	18.51	25.76	29.03	28.95	23.85	**39.30**
	BSF	1.00	2.27	8.73	3.20	1.13	**10.70**
HIP 11767	SCR (dB)	33.77	34.81	30.12	35.48	36.84	**37.35**
	SNR (dB)	29.29	34.31	39.12	37.38	33.73	**44.32**
	BSF	1.00	1.92	6.31	2.59	1.03	**5.69**
HIP 24608	SCR (dB)	26.81	27.22	29.78	29.68	27.82	**39.91**
	SNR (dB)	21.82	27.11	30.15	30.34	25.55	**37.01**
	BSF	1.00	1.92	6.73	2.68	1.04	**6.17**

Note: Bold values correspond to the results of the proposed method.

**Table 3:** Comparison of computational efficiency and speedup.

Algorithm	Average Time (s)	Speedup
RPCA	1.37	1×
Top-Hat	0.007	176.1×
LoG	0.002	616.5×
JLCM	0.014	88.1×
Proposed	0.16	8.56×

**Table 4:** Ablation study on the base decomposition framework and structural weighting strategies.

Image	Metric	Original	Unweighted	Gaussian Weighted	Proposed
HIP 28380	SCR (dB)	10.35	Failed	11.01	**11.62**
	SNR (dB)	8.48	Failed	16.37	**22.69**
	BSF	1.00	17.68	3.14	**5.88**
HIP 50335	SCR (dB)	21.18	Failed	29.17	**33.99**
	SNR (dB)	15.97	Failed	29.75	**35.34**
	BSF	1.00	24.15	4.83	**9.41**
HIP 50583	SCR (dB)	25.20	164.17	37.69	**39.47**
	SNR (dB)	18.51	−8.06	32.62	**39.30**
	BSF	1.00	27.38	4.95	**10.70**
HIP 11767	SCR (dB)	33.77	217.35	37.29	**37.35**
	SNR (dB)	29.29	44.62	39.08	**44.32**
	BSF	1.00	13.79	3.12	**5.69**
HIP 24608	SCR (dB)	26.81	Failed	38.57	**39.91**
	SNR (dB)	21.82	Failed	31.75	**37.01**
	BSF	1.00	17.86	3.34	**6.17**

Note: Bold values correspond to the results of the proposed method. In certain sequences (e.g., HIP 50583, HIP 11767), the extremely high SCR values observed in the unweighted model are numerical artifacts. Because global soft-thresholding over-smooths the image, the local background standard deviation (the denominator in SCR calculation) artificially approaches zero. This severely exaggerates the SCR score, even though the actual target signal has already degraded or been lost.

## Data Availability

The raw data supporting the conclusions of this article will be made available by the authors on request.

## Abbreviations

The following abbreviations are used in this manuscript:

SWIR	Short-Wave Infrared
PSF	Point Spread Function
WLRSD	Weighted Low-Rank Sparse Decomposition
BRP	Bilateral Random Projection
RPCA	Robust Principal Component Analysis
IALM	Inexact Augmented Lagrange Multiplier
JLCM	Joint Local Contrast Measure
LoG	Laplacian of Gaussian
SNR	Signal-to-Noise Ratio
SCR	Signal-to-Clutter Ratio
BSF	Background Suppression Factor
